# Diverse genetic architectures lead to the same cryptic phenotype in a yeast cross

**DOI:** 10.1038/ncomms11669

**Published:** 2016-06-01

**Authors:** Matthew B. Taylor, Joann Phan, Jonathan T. Lee, Madelyn McCadden, Ian M. Ehrenreich

**Affiliations:** 1 Molecular and Computational Biology Section, Department of Biological Sciences, University of Southern California, Los Angeles, California 90089, USA

## Abstract

Cryptic genetic variants that do not typically influence traits can interact epistatically with each other and mutations to cause unexpected phenotypes. To improve understanding of the genetic architectures and molecular mechanisms that underlie these interactions, we comprehensively dissected the genetic bases of 17 independent instances of the same cryptic colony phenotype in a yeast cross. In eight cases, the phenotype resulted from a genetic interaction between a *de novo* mutation and one or more cryptic variants. The number and identities of detected cryptic variants depended on the mutated gene. In the nine remaining cases, the phenotype arose without a *de novo* mutation due to two different classes of higher-order genetic interactions that only involve cryptic variants. Our results may be relevant to other species and disease, as most of the mutations and cryptic variants identified in our study reside in components of a partially conserved and oncogenic signalling pathway.

## Abstract (web summary)

Cryptic genetic variants may not individually show discernible phenotypic effects, but collectively, these polymorphisms can lead to unexpected, genetically complex traits that might be relevant to evolution and disease. Here, the authors use large yeast populations to comprehensively dissect the genetic bases of 17 independent occurrences of a phenotype that arises due to combinations of epistatically interacting cryptic variants.

## Graphical abstract

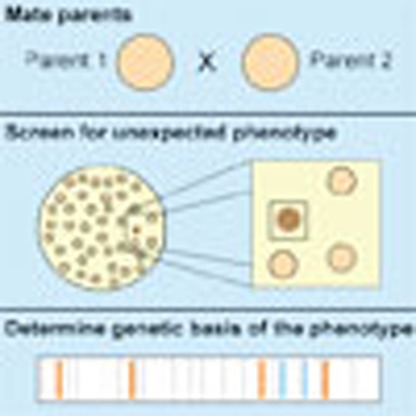

Cryptic genetic variants are standing polymorphisms that only show phenotypic effects under atypical conditions, such as when specific genes are mutated, rare combinations of segregating alleles are generated, or the environment markedly changes^1^,^2^,^3^,^4^,^5^,^6^,^7^,^8^. Given a stable environment, the uncovering of cryptic variation can be viewed as a form of epistatic (or genetic) interaction, in which the phenotypic effects of cryptic variants depend on the mutations and standing polymorphisms with which they co-occur^9^,^10^,^11^. Empirical and theoretical evidence suggests that these epistatic interactions may involve large numbers of loci that do not individually influence phenotype, but collectively exhibit significant trait effects (so-called ‘higher-order genetic interactions')^9^,^11^,^12^,^13^,^14^,^15^. However, the genetic architectures and molecular mechanisms that underlie these epistatic interactions have yet to be characterized in detail^16^.

We have developed an experimental system in the budding yeast *Saccharomyces cerevisiae* that provides a powerful resource for identifying and studying epistatic interactions among cryptic variants and mutations^11^,^13^,^17^. Specifically, under the standard temperature used for culturing yeast (30 °C), the lab strain BY4716 (‘BY')^18^, a haploid derivative of the clinical isolate 322134S (‘3S')^18^, and their wild-type recombinant progeny show a ‘smooth' colony phenotype^13^. However, induced and spontaneous mutations can cause BY × 3S recombinants possessing particular combinations of segregating cryptic variants to exhibit an alternative, ‘rough' colony morphology^11^,^13^,^17^ (Fig. 1a). Because the rough phenotype is only expressed when epistatic interactions among cryptic variants and mutations occur, the phenotype can be used as a reporter to detect these interactions. Furthermore, once an individual rough segregant is obtained, the specific combinations of cryptic variants and mutations that cause this individual to express the phenotype can be determined through genetic mapping in backcrosses (Fig. 1b).

In the most thoroughly characterized example of the rough phenotype's genetic basis, we demonstrated that a *de novo* frameshift mutation in the Ras negative regulator *IRA2* (*ira2Δ*2933) can alter colony morphology when it co-occurs with one of two specific combinations of cryptic variants in six genes^11^,^13^,^17^. In more recent work, we showed that the genetic architectures that enable *ira2Δ*2933 to induce the rough phenotype vary across temperatures^17^. For instance, a single, specific combination of seven cryptic variants facilitates *ira2Δ*2933-dependent rough morphology at 37 °C. However, at room temperature (21 °C), two BY × 3S *ira2Δ*2933 multi-locus genotypes—one involving two cryptic variants and the other involving six cryptic variants—show rough morphology. Moreover, some BY × 3S segregants appear to express the phenotype at 21 °C independent of any mutation^17^. These results suggest that, out of the three temperatures we have previously examined, the greatest diversity of genetic architectures underlying the rough phenotype occurs at 21 °C.

Here we take advantage of the rough colony morphology system to conduct the first large-scale examination of epistatic interactions among cryptic variants and *de novo* mutations. To perform this work, we screen >100 independent BY × 3S F_2_ crosses for the rough phenotype at 21 °C. Through this screen, we obtain 17 independent occurrences of the trait. We then use genetic mapping in backcrosses to comprehensively identify genetic factors that contribute to each instance of the rough phenotype. In addition, we clone the specific genes underlying most of the detected loci, thereby obtaining new insights into the molecular mechanisms that uncover cryptic variation.

On the basis of these efforts, we find that the genetic architectures that can lead to the rough phenotype at 21 °C are quite diverse. About half of the instances of the rough phenotype represent epistatic interactions among cryptic variants and *de novo* mutations. These cases involve six different mutated genes, as well as between one and nine cryptic variants. The remaining instances of the rough phenotype do not require *de novo* mutations and instead arise due to higher-order genetic interactions that only involve cryptic variants. These mutation-independent cases fall into two classes that are distinguished by the occurrence of recombination within a specific ∼1.3-kb genomic interval in the promoter of a required cell surface protein. We also demonstrate that the vast majority of genetic factors involved in our study influence Ras signalling or Ras-dependent transcriptional regulation. Thus, our results not only shed light on the forms of epistatic interactions that can enable cryptic variants to influence phenotype, but also implicate complex changes in gene regulation in a partially conserved and disease-associated signalling pathway as the main source of these interactions.

## Results

### Genetic mapping of 17 independent cases of rough morphology

**Figure 1:**
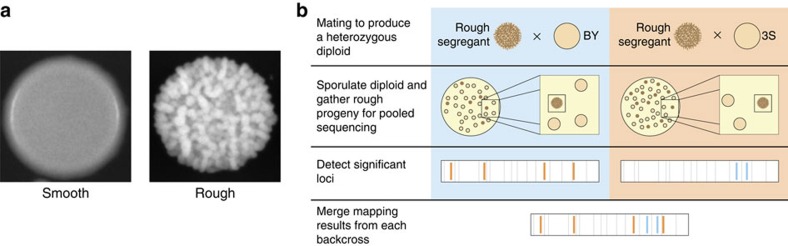
Overview of study. (**a**) Representative images of rough and smooth colonies in the BY × 3S cross. (**b**) Backcrossing strategy used to determine the genetic basis of the rough phenotype in a given F_2_ segregant.

We mated BY and 3S 106 independent times, and screened >100,000 haploid F_2_ segregants derived from these matings at 21 °C (Methods). Through this screen, we obtained 17 rough segregants that were descended from different BY/3S diploids and thus represented biologically independent occurrences of the same phenotype (Supplementary Fig. 1; Methods). To determine the genetic bases of these distinct instances of the rough phenotype, we backcrossed each rough segregant to both BY and 3S. Bulk segregant mapping by sequencing^19^,^20^,^21^ was performed on pools containing between 46 and 95 rough F_2_B progeny (Fig. 1b; Supplementary Table 1). These pools of rough backcross segregants were sequenced to an average genomic coverage of 246 × (Methods). Control pools were also generated for each backcross using lawns containing millions of random progeny that had not been selected based on their colony morphologies (Methods). These control pools were sequenced to an average genomic coverage of 162 × (Methods).

**Figure 2:**
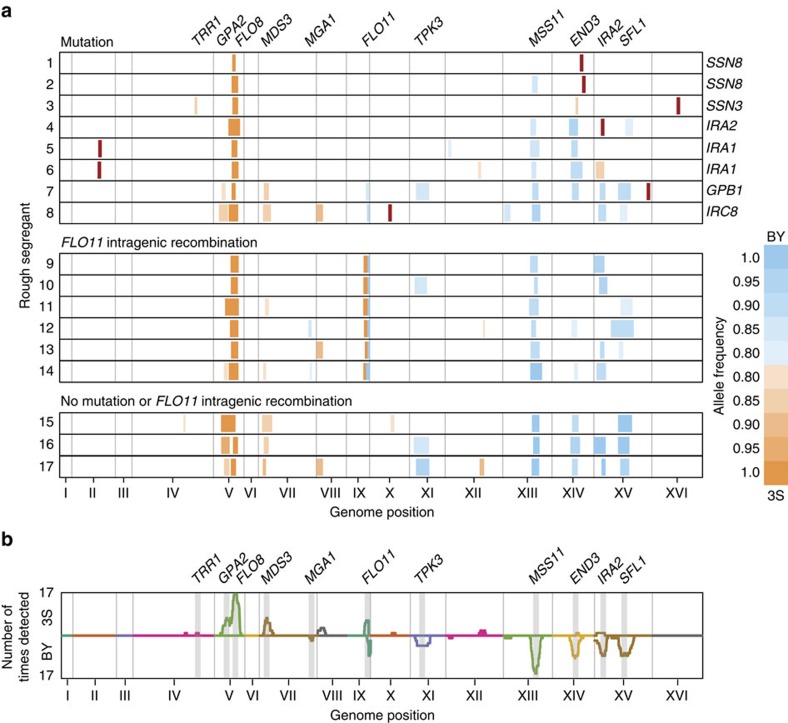
Genetic mapping results. (**a**) Genetic mapping data for each rough segregant is shown horizontally. The mapping results correspond to data from backcrosses of each segregant to BY and 3S, as depicted in Fig. 1b. Vertical bars represent detected loci, with blue and orange colouring indicating cryptic variants from BY and 3S, respectively. The width of each locus corresponds to the region of that locus that exhibits a logarithm of odds score of at least 5. *De novo* mutations are shown in red, with the specific mutated gene noted to the right of the panel. The allele frequencies of detected loci in a given backcross mapping population are provided, with the colour scale illustrated to the right of the figure. (**b**) The number of times each cryptic variant was detected across the different mapping populations is plotted. Counts were determined by summing the number of times each segregating marker was detected in backcrosses to a particular parent. Results corresponding to *de novo* mutations were excluded.

We separately analysed the rough and control populations for each backcross using MULTIPOOL^22^ and excluded any loci detected in the control pools from further consideration (Methods; Supplementary Table 2; Supplementary Table 3). On the basis of this procedure, we identified between 2 and 10 loci per rough segregant, with an average of 6.6 loci (Supplementary Table 4). These detected genomic regions corresponded to 8 *de novo* mutations and 18 distinct segregating loci that harbour cryptic variants (Fig. 2a,b; Methods; Supplementary Fig. 2). Only one of these cryptic variants, which we previously localized to the 3S version of the Ras-regulated transcriptional activator *FLO8* (ref. 13), was fixed in all our genetic mapping experiments (Fig. 2a,b). Given that BY carries a null allele of *FLO8* (refs 23, 24), this finding indicates that a functional copy of *FLO8* is necessary for the phenotype's expression. The remaining loci were detected on average 4.1 times, with a range between 1 and 15.

### Genes that can lead to rough morphology when mutated

The eight *de novo* lesions were comprised of six small deletions and two point mutations (Supplementary Fig. 3; Supplementary Table 5). The six genes harbouring these changes fell into three functional classes (Supplementary Table 5): negative regulators of Ras signalling (*GPB1*, *IRA1* and *IRA2*), non-essential components of RNA polymerase II that act in the mediator complex (*SSN3* and *SSN8*) and a gene of unknown function whose product localizes to bud tips during cell division (*IRC8*)^25^. On the basis of gene deletion experiments (Methods), seven on the mutations were found to be null alleles (Supplementary Fig. 4). The only partial loss-of-function mutation was a point mutation in *GPB1* (Supplementary Fig. 4; Supplementary Table 5). This is consistent with our previous finding that *ira2Δ*2933 is also a partial loss-of-function allele^13^, as Gpb1 and Ira2 physically interact to coregulate Ras signalling, and these lesions in *GPB1* and *IRA2* fall within (*GPB1*) or truncate (*IRA2*) protein–protein interaction domains needed for Gpb1–Ira2 binding^26^ (Supplementary Table 5).

When considered with our previous work, in which *ira2Δ*2933 and complete knockout of the Ras-regulated transcriptional repressor *SFL1* were shown to reveal the rough phenotype^11^,^13^, we have now identified seven genes with the potential to alter colony morphology when mutated. The identities of these genes, as well as the fact that the mediator complex is regulated by Ras signalling^27^ and physically interacts with Sfl1 to inhibit transcription^28^, suggests that most of the mutations we have identified influence transcriptional regulation by the Ras pathway. This finding supports our recent discovery that expression of the rough phenotype in the BY × 3S cross requires transcriptional derepression of one or more Ras target genes^11^. However, even though the identified mutations each likely lead to derepression, they show significant differences in the combinations of cryptic variants they interact with to exert their effects. Specifically, between one and nine cryptic variants were detected in backcross populations derived from the mutants (Fig. 2a; Supplementary Table 4). As we discuss later, these differences in genetic complexity among rough segregants may relate to the rough phenotype's underlying gene regulatory network.

### Genetics of mutation-independent rough colony morphology

**Figure 3:**
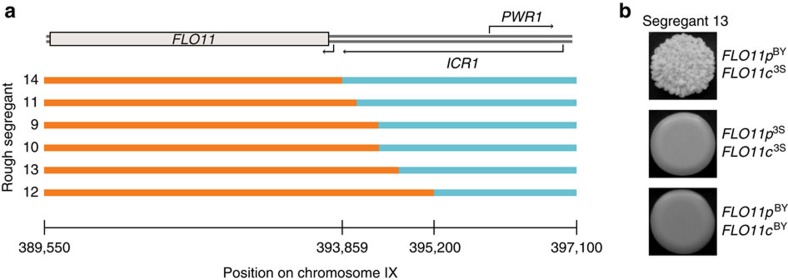
Locations of independent recombination events within the *FLO11* locus. (**a**) Genotypes of rough segregants that possess a *FLO11* intragenic recombination event are shown, along with annotations of this locus from the *Saccharomyces* Genome Database^25^. *ICR1* and *PWR1* encode noncoding RNAs. BY and 3S segments are shown in blue and orange, respectively. (**b**) Representative images of allele replacements in the coding (‘c') and promoter (‘p') regions of *FLO11*, which verify the presence of at least two cryptic variants in different regions of this gene, are provided.

The nine other rough segregants did not harbour *de novo* mutations. This supports a previous finding that some individuals in the BY × 3S cross may show rough morphology despite lacking *ira2Δ*2933 or other mutations^17^. Five or more loci were detected in each of these cases, implying they occurred due to higher-order genetic interactions that only involve cryptic variants. All of the mapping populations lacking mutations were fixed for *MSS11*^BY^, which encodes an activator that heterodimerizes with Flo8 (refs 29, 30). Two-thirds of these individuals also possessed intragenic recombinations within a ∼1.3-kb region preceding the transcription start site of *FLO11*, which encodes a cell surface glycoprotein that must be transcribed for the rough phenotype to be expressed^11^ (Figs 2a and 3a). Specifically, the BY promoter and the 3S coding region of *FLO11* harbour cryptic variants that together enable expression of the rough phenotype in the presence of other cryptic variants that segregate in the BY × 3S cross (Fig. 3b). The remaining third of the wild-type rough segregants did not exhibit dependence on particular *FLO11* haplotypes. Instead, mapping data for these cases consistently showed enrichment for cryptic variants on chromosomes V, VII, XIV and XV, as well as other loci that differed among the individuals (Fig. 2a).

### Most identified cryptic variants affect the Ras pathway

**Figure 4:**
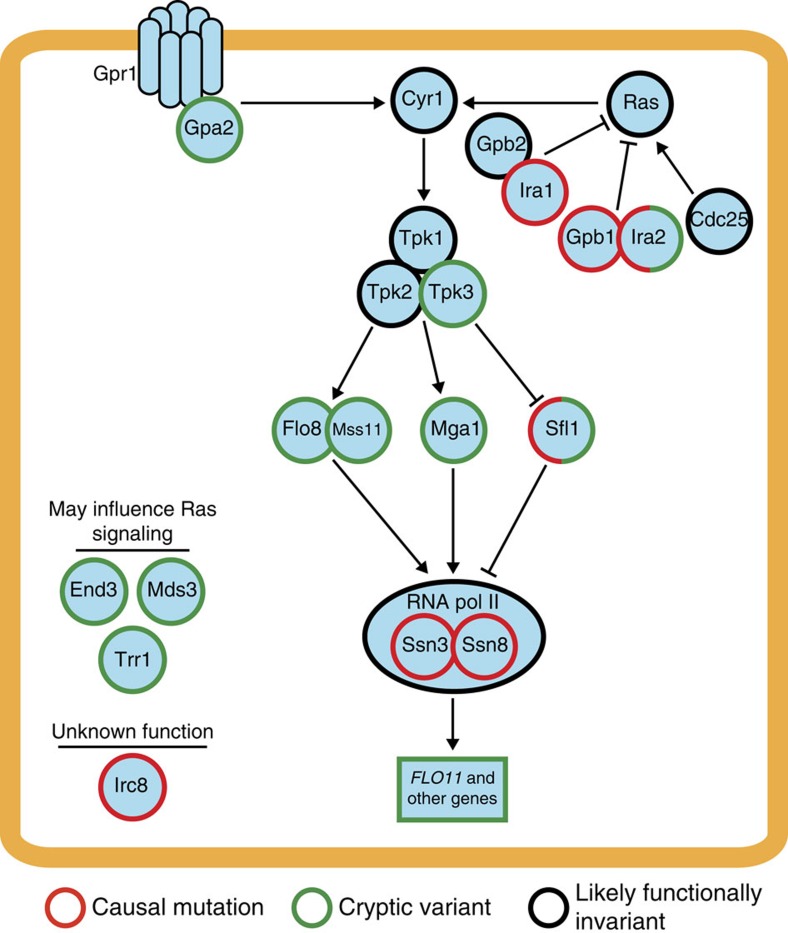
Genes involved in the rough phenotype in the BY × 3S cross largely regulate signalling and transcriptional control by the Ras pathway. A simplified portrait of the Ras pathway is presented. Components of the Ras pathway and other identified proteins are colour coded by whether they can result in the rough phenotype when mutated (red), harbour cryptic variants (green) or are unlikely to harbour functionally distinct alleles that affect colony morphology in the cross (black). *IRA2* and *SFL1* are coloured as both green and red because they fall into two of the aforementioned classes. Most of the genes that uncover the rough phenotype when mutated or possess cryptic variants function in Ras signalling and Ras-dependent transcriptional regulation. Certain proteins, namely End3, Mds3 and Trr1, do not act directly in the Ras pathway, but have been shown to either influence or be influenced by the Ras activity. Our results also suggest that Irc8 might have some functional relationship to the Ras pathway.

We next sought to define the specific genes that harbour cryptic variation and found that most are involved in Ras signalling or Ras-dependent transcriptional regulation. Among the 16 loci not corresponding to the *FLO11* promoter or coding region, seven contain genes that we previously showed to harbour cryptic variation or be capable of uncovering the rough phenotype when mutated^11^,^13^,^17^. In addition to *FLO8*, *IRA2*, *MSS11* and *SFL1*, we detected loci overlapping the vesicle component *END3*, the activator *MGA1* and the redox stress detoxifier *TRR1* (Fig. 2a,b). As with Flo8–Mss11 heterodimer and Sfl1, Mga1 acts downstream of the Ras pathway and regulates *FLO11* and other genes that are important for yeast colony morphology traits^31^ (Fig. 4). Furthermore, although End3 and Trr1 are not components of the Ras pathway, functional relationships between these genes and Ras signalling likely exist^32^,^33^. We also cloned the causal genes underlying three loci that had not previously been characterized. By performing allele replacements in multiple rough segregants, we successfully resolved loci on chromosome V, VII and XI to *GPA2*, *MDS3* and *TPK3*, respectively (Fig. 2a,b; Supplementary Fig. 5; Methods). These genes encode a G protein subunit that is required for the recruitment of Ras-GTP (*GPA2*)^25^, a component of the target of rapamycin pathway that has also been shown to influence Ras signalling (*MDS3*)^34^, and a subunit of the Ras effector kinase protein kinase A (*TPK3*)^25^.

## Discussion

In summary, we have determined the genetic architectures underlying 17 independent instances of the same cryptic phenotype. These different occurrences of rough colony morphology vary significantly in their numbers of involved cryptic variants and also in whether they require a *de novo* mutation. Among cases involving *de novo* mutations, our work suggests that the rough phenotype's genetic architecture depends on the gene that is mutated. This relationship is likely tied to Ras signalling and the transcriptional control of Ras target genes (Fig. 4). For example, Ssn3 and Ssn8 act the most proximally to transcription, and rough *SSN3* and *SSN8* mutants show the lowest genetic complexity in our study (Figs 2a and 4). In contrast, Gpb1, Ira1 and Ira2 act in the upstream portion of the Ras cascade, and rough *GPB1*, *IRA1* and *IRA2* mutants exhibit relatively high numbers of detected loci (Figs 2a and 4). Furthermore, the *IRC8* mutant also shows a high number of detected loci (Fig. 2a), suggesting this uncharacterized gene might also act upstream of the Ras pathway.

In addition, our study illustrates how the rough phenotype can arise in certain environments, here 21 °C, due to higher-order genetic interactions that only involve cryptic variants (Fig. 2a). These combinations of cryptic variants presumably recapitulate the molecular and systems level effects of epistatic interactions that involve both cryptic variants and *de novo* or induced mutations^11^,^13^,^17^. This scenario is supported by the fact that certain genes, namely *IRA2* and *SFL1*, appear to both possess cryptic variants and have the potential to uncover the rough phenotype when mutated (Fig. 4). Tied to this point, we note that while *GPB1*, *IRA1*, *IRC8*, *SSN3* and *SSN8* do not seem to harbour cryptic variation in the BY × 3S cross, it is possible that other *S. cerevisiae* strains carry cryptic variation in these genes.

Given that most of the genes involved in the rough phenotype act in or are influenced by the Ras pathway (Fig. 4), which has components that are evolutionarily conserved^35^, our findings might extend to other species and traits. In fact, cryptic variation in the Ras pathway is known to impact the development in *Caenorhabditis elegans*^36^, and perturbation of Ras pathway components in humans can lead to cancer and other diseases^37^. Thus, further characterizing cryptic variation in the Ras pathway in yeast might provide valuable new insights into the mechanisms that give rise to genetically complex phenotypes, which are relevant to health and evolution.

## Methods

### Phenotyping of yeast colony morphology

Strains were grown at 30 °C overnight in liquid media comprised of yeast extract and peptone (YP) with 2% dextrose as the carbon source (YPD). Stationary-phase cultures were pinned onto YP agar media with 2% ethanol as the carbon source (YPE) and grown for 7 days at 21 °C, unless otherwise noted.

### Generation of rough segregants

The rough segregants examined in this work come from a cross between BY4716 (`BY'), a descendant of the reference strain S288c, and a haploid derivative of 322134S (`3S'), a clinical isolate. Strains used in this work possessed the synthetic genetic array (SGA) marker system^38^, which facilitates rapid generation of *MAT**a*** haploid progeny from diploid strains. This system allows for selection of *MAT**a*** spores by plating of sporulated cultures onto yeast nitrogen base (YNB) media containing canavanine. To generate a diploid, a *MAT***a** BY strain and a *MATα* 3S strain were grown overnight in YPD, and then mixed on YPD agar media and incubated for 4 h at 30 °C. Microdissection was then used to obtain diploid BY/3S zygotes. This process was repeated 106 independent times. A single diploid from each mating was sporulated as in Taylor and Ehrenreich (2014)^13^, after which segregants were plated onto YNB containing canavanine to an average density of ∼200 per plate. In total, >500 such plates and >100,000 segregants were produced. Once colonies were visible on these YNB plates, they were replicated onto YPE plates. After 7 days of growth at room temperature, segregants were screened for rough morphology. To ensure that all instances of the rough phenotype were biologically independent, no more than one rough segregant was gathered from any particular diploid.

### Generation of backcross segregants

Each rough segregant was backcrossed to both BY and 3S to generate diploids in the same manner described above. These backcross diploids were sporulated and then plated onto YNB media containing canavanine to select for *MAT**a*** haploid progeny. Between 46 and 95 rough segregants were obtained from each backcross (Supplementary Table 1). Rough segregants from each backcross were grown to stationary phase in YPD at 30 °C. These stationary cultures were then mixed together in equal volumes and DNA was extracted from these pools of rough segregants using the Qiagen Genomic-tip 100/G kit. To generate control populations for these backcrosses, sporulations were plated at high density onto YNB with canavanine and grown at 21 °C for 2 days to produce lawns containing millions of random backcross segregants. These lawns were scraped directly off the plates and DNA was extracted from these pools of cells, using the Qiagen Genomic-tip 100/G kit.

### Sequencing of mapping populations

Sequencing libraries were generated from each backcross pool, using the Illumina Nextera kit. These libraries were sequenced on an Illumina NextSeq machine by the USC Epigenome Center, using 75 × 75 base reads. Rough pools were sequenced to an average of 246-fold and control pools were sequenced to an average of 162-fold coverage. Reads were aligned to either a BY or 3S reference genome, using the Burrows–Wheeler Aligner version 7 with option mem −t 20 (ref. 39) and mpileup files were generated with SAMtools^40^. Sequencing data can be accessed from the Sequence Read Archive using Biosample accession numbers SAMN04126845 through SAMN04126912 or the Bioproject accession number PRJNA301897. The specific accession number for each pool is listed in Supplementary Table 6.

### Genetic mapping using MULTIPOOL

Genome-wide allele frequencies at 36,756 high-confidence single-nucleotide polymorphisms were determined by a custom Python script^11^,^13^. Loci were detected in each mapping population using MULTIPOOL^22^ with settings: replicates mode, 3,300-bp centimorgans and 100-bp bins. Segregating genomic intervals in each population were independently analysed, with a region considered significant in a given backcross if it had a logarithm of odds (LOD) score ≥5 across a region of at least 30 kb. We considered the span of each locus as the 90% confidence interval surrounding the point of maximal significance, as determined by MULTIPOOL. Loci that were detected in control backcross populations were ignored from their corresponding populations of rough segregants. Also, detected loci that were within 50 kb of the mating locus were excluded, as the mating locus is used in the SGA marker system. All loci identified in mapping and control experiments can be found in Supplementary Tables 3 and 4. In a few instances, detected loci were broad and overlapped two cryptic variants that were previously identified^11^,^13^,^17^ or were cloned in the current study. In such cases, these broad regions were counted as two loci.

### Identification of *de novo* mutations

Sequence data for the mapping populations were examined for genetic differences relative to the BY and 3S reference genomes, using a combination of custom Python scripts and manual inspection. Identified lesions were then validated by PCR and Sanger sequencing of a potentially mutated site in the corresponding rough segregant. Primers for these sequencing experiments are provided in Supplementary Table 7. In addition, we investigated the role of chromosomal anomalies, such as aneuploidies, inversions and translocation, and did not see evidence of such events playing a role in the phenotype (Supplementary Note 1).

### Genetic engineering experiments

Allele replacements were performed using a modified form of adaptamer-mediated allele replacement^11^,^24^,^41^. Transformations were conducted with two partially overlapping PCR products—a full-length amplicon of a gene of interest that was tailed at the 3′ end, with the 5′ portion of the *kanMX* cassette and a copy of the *kanMX* cassette that was tailed on the 3′ end, with part of the intragenic region downstream of the gene^11^,^24^. Knock-ins were identified using selection on G418 and verified by Sanger sequencing. At least three knock-in strains were screened per allele replacement. Gene deletions were performed by replacing a gene of interest with the CORE cassette^42^. Regions corresponding to 60 bases upstream and downstream of the target gene were tailed to the CORE cassette using PCR. This product was transformed into cells using the lithium acetate method^43^, and selection with G418 was used to screen for integration of the cassette. PCR was then used to verify that deletion strains recovered from the G418 selection lacked the gene of interest. Primers used in genetic engineering experiments can be found in Supplementary Table 7.

### Data availability

All sequencing data has been deposited in the National Center for Biotechnology Information (NCBI) Short Read Archive, and can be accessed under the BioProject ID PRJNA301897 and BioSample accessions SAMN04126845–SAMN04126912. A full list of accessions can be found in Supplementary Table 6.

## Additional information

**How to cite this article:** Taylor, M. B. *et al*. Diverse genetic architectures lead to the same cryptic phenotype in a yeast cross. *Nat. Commun.* 7:11669 doi: 10.1038/ncomms11669 (2016).

## Supplementary Material

Supplementary Information Supplementary Figures 1-5, Supplementary Tables 1-7, Supplementary Notes 1 and Supplementary References
