# Supplementary material for: Diverse genetic architectures lead to the same cryptic phenotype in a yeast cross
Source: Nat Commun. 2016 Jun 1;7:11669. doi: 10.1038/ncomms11669 (PMC4895441; doi:10.1038/ncomms11669)
Supplement: Supplementary Information — Supplementary Figures 1-5, Supplementary Tables 1-7, Supplementary Notes 1 and Supplementary References [file ncomms11669-s1.pdf]

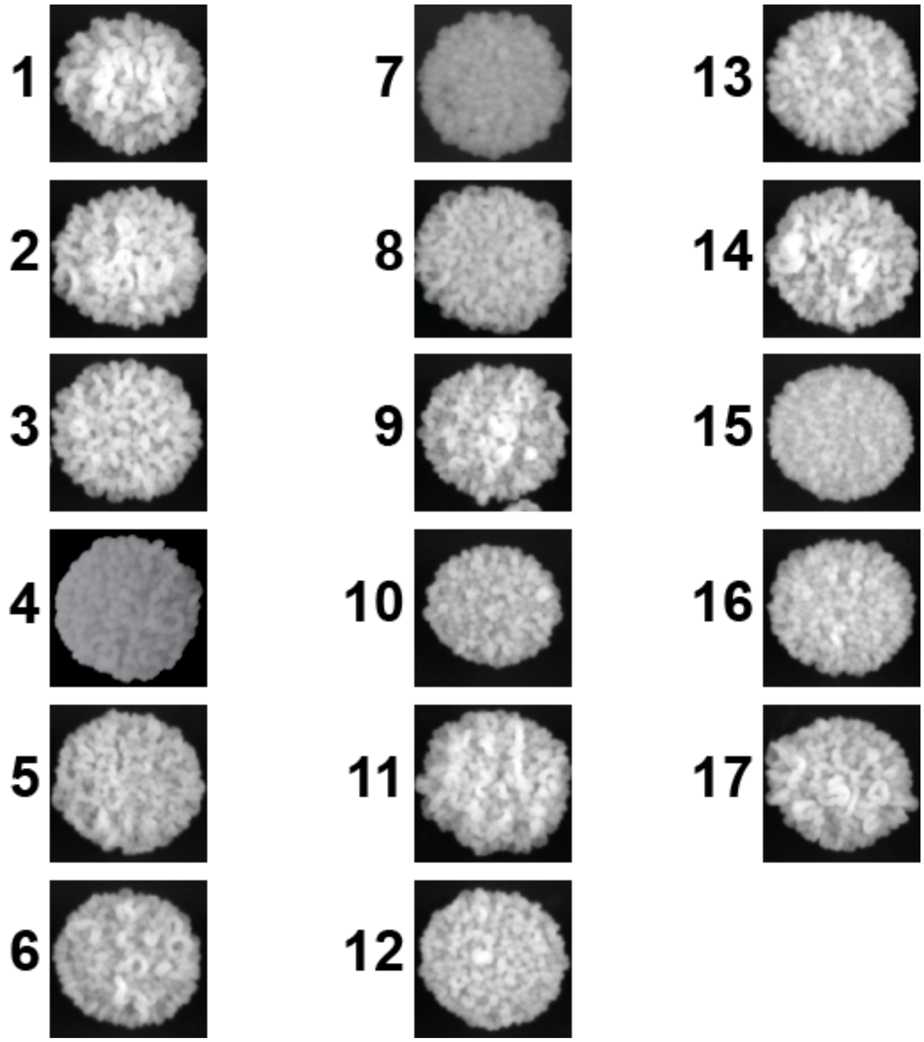

Supplementary Figure 1. Phenotypes of the 17 identified rough segregants at 21ºC.

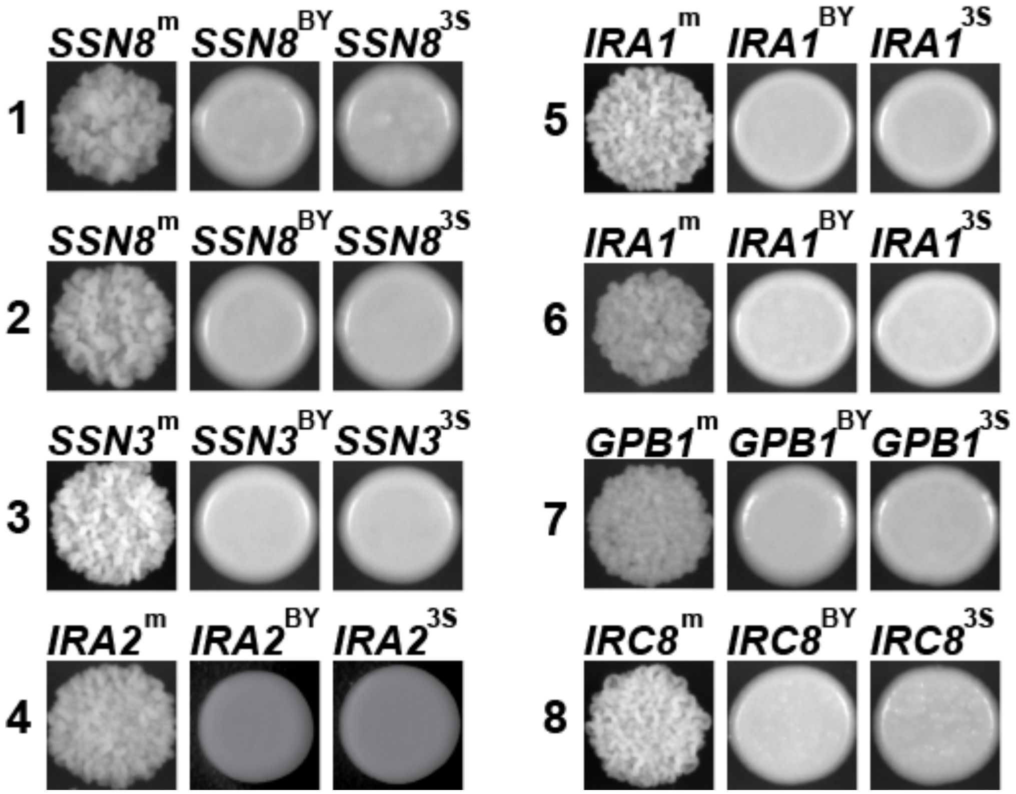

Supplementary Figure 2. Verification that identified *de novo* mutations have phenotypic effects in rough segregants 1 through 8. Allele replacements were performed in the rough segregant in which a *de novo* mutation was detected. For a gene harboring a *de novo* mutation in a given rough segregant, we performed three genetic engineerings. The mutant allele was replaced with itself, the wild type BY allele, and the wild type 3S allele. These replacements are referred to in superscript as ‘m’, ‘BY’, and ‘3S’, respectively. For each of the eight *de novo* mutations, replacement of the lesion with wild type BY or 3S alleles resulted in a conversion from rough to smooth colony morphology. This implies that the *de novo* mutations play causal roles in a given individual’s expression of the rough phenotype.

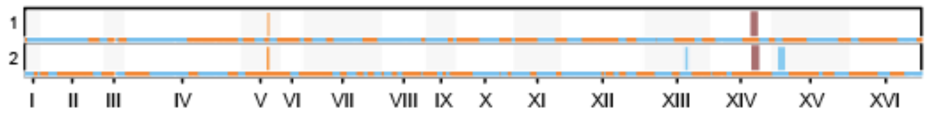

Supplementary Figure 3. Rough segregants 1 and 2 possess the same *SSN8* lesion, but are likely distinct individuals obtained from different matings of BY and 3S. Genome-wide haplotypes are shown below mapping data from Fig. 2a. Blue horizontal bars represent regions in which the segregant possessed the BY allele and orange horizontal bars represent regions in which the segregant possessed the 3S allele. The red vertical bars indicate the *SSN8* mutations. As can be seen from their haplotypes, rough segregants 1 and 2 do not share any recombination breakpoints, indicating they are not spores from the same tetrad. Because each rough segregant was obtained from independent matings of BY and 3S, it is unlikely that these individuals' *SSN8* lesions arose in the same mutational event.

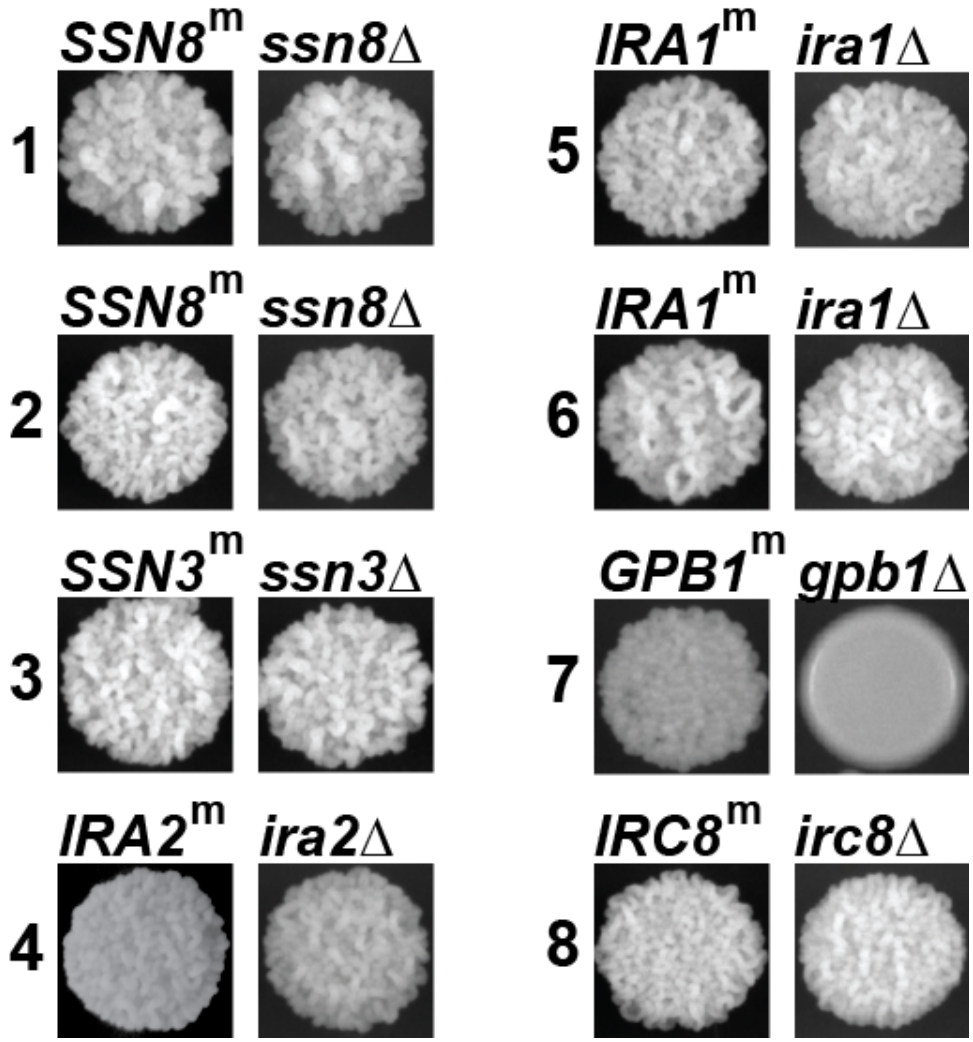

Supplementary Figure 4. Complete deletion of mutated genes from rough segregants 1 through 8. To test whether the identified *de novo* mutations were null or partial loss-of-function alleles, we deleted these genes in their entirety from the corresponding rough segregant. Lack of effect of these complete deletions on colony morphology indicates that a given *de novo* mutation is likely a null allele. In contrast, a conversion from rough to smooth colony morphology suggests that a particular *de novo* mutation is a partial loss-of-function allele.

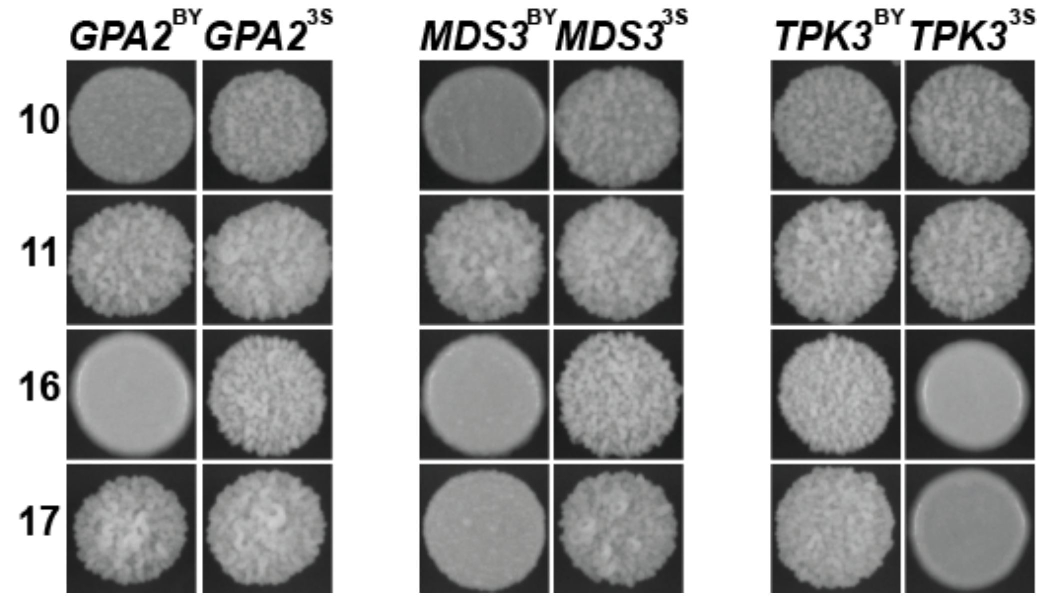

Supplementary Figure 5. Allele replacements verify that *GPA2*^{3S}, *MDS3*^{3S}, and *TPK3*^{BY} harbor cryptic variants with genetic background-dependent effects. We replaced the causal allele of a given gene with the alternative allele in four different rough segregants. As a control, causal alleles were also replaced with themselves. Representative allele replacement results are shown for each allele replacement experiment in each rough segregant. Please note that the pictures are ordered so that allele replacements involving the BY and 3S alleles are always shown to the left and right, respectively.

Supplementary Table 1. Number of individuals in each pooled sequencing experiment.

RS1xBY	92
RS1x3S	95
RS2xBY	84
RS2x3S	83
RS3xBY	82
RS3x3S	92
RS4xBY	95
RS4x3S	86
RS5xBY	93
RS5x3S	90
RS6xBY	85
RS6x3S	89
RS7xBY	84
RS7x3S	48
RS8xBY	92
RS8x3S	52
RS9xBY	69
RS9x3S	69
RS10xBY	89
RS10x3S	68
RS11xBY	86
RS11x3S	74
RS12xBY	90
RS12x3S	53
RS13xBY	88
RS13x3S	72
RS14xBY	90
RS14x3S	74
RS15xBY	69
RS15x3S	46
RS16xBY	70
RS16x3S	50
RS17xBY	48
RS17x3S	70

Supplementary Table 2. Loci detected in rough backcross pools. Start and stop positions were called as the 90% confidence interval around the point of maximum significance. Allele frequencies were calculated as the maximum within a 25-SNP window within a locus.

Segregant	Cross	Chromosome	Start	Stop	Frequency
1	3S	3	157990	235590	0.99
1	3S	5	6639	64439	1.00
1	BY	5	349582	413382	0.99
1	BY	14	458048	649748	1.00
2	BY	3	91793	147893	0.84
2	3S	3	175351	212951	0.99
2	3S	5	6639	89139	1.00
2	BY	5	337336	461436	0.99
2	3S	13	547996	652396	0.87
2	BY	14	504670	686470	1.00
3	3S	3	78378	217178	0.99
3	BY	4	1172541	1220841	0.85
3	3S	5	5035	352535	1.00
3	BY	5	353654	460054	1.00
3	BY	14	441083	488583	0.86
3	3S	16	427572	563072	1.00
4	3S	3	90850	228750	1.00
4	BY	4	1173122	1259022	0.87
4	3S	5	1435	79335	0.99
4	BY	5	277713	497113	1.00
4	3S	13	514890	620490	0.88
4	3S	14	319549	485449	0.98
4	3S	15	88326	237926	0.99
4	3S	15	586426	733426	0.81
5	3S	2	450356	602056	1.00
5	3S	3	92377	219377	1.00
5	3S	5	6535	64435	1.00
5	BY	5	336513	446913	1.00
5	3S	12	56412	118112	0.80
5	3S	13	510149	686349	0.96
5	3S	14	360204	480404	0.95
6	BY	2	481372	547972	1.00
6	3S	2	548339	657239	0.82
6	3S	3	105147	207047	1.00
6	3S	5	1435	81635	1.00

6	BY	5	341045	467745	0.99
6	BY	12	617236	672536	0.82
6	3S	13	534632	626532	0.88
6	3S	14	350144	570944	0.90
6	BY	15	34977	191977	0.88
7	3S	3	132313	219813	0.98
7	3S	5	1435	70235	0.99
7	3S	9	366656	418556	0.87
7	3S	11	119772	366972	0.88
7	3S	13	552176	667676	0.96
7	3S	14	373335	499935	0.91
7	BY	15	107986	215386	0.07
7	3S	15	451598	689798	0.92
8	3S	3	112256	214056	0.99
8	3S	5	28135	78135	1.00
8	BY	5	101481	271781	0.87
8	BY	5	286081	461781	0.99
8	BY	7	85900	234000	0.88
8	3S	8	441	142341	0.07
8	3S	9	383751	432851	0.95
8	3S	10	299283	464883	0.99
8	3S	13	25332	140232	0.83
8	3S	13	545805	702505	0.98
8	3S	15	82512	228312	0.92
8	3S	15	486712	624312	0.84
9	3S	3	97868	220168	0.99
9	3S	5	6639	69839	0.98
9	BY	5	319074	470274	1.00
9	BY	9	320560	394160	0.99
9	3S	9	395108	434008	0.99
9	3S	13	511018	655418	0.94
9	3S	15	126	197426	0.95
10	3S	3	105083	218983	1.00
10	BY	4	1169284	1269984	0.92
10	3S	5	4635	71335	0.99
10	BY	5	323425	456025	0.99
10	BY	9	317767	393767	1.00
10	3S	9	395108	432108	0.98
10	3S	11	94872	326772	0.86
10	3S	13	584908	654908	0.92
10	3S	15	95414	250914	0.98

11	3S	3	69577	232977	1.00
11	3S	5	1935	73635	1.00
11	BY	5	215722	472022	1.00
11	BY	7	124322	195022	0.81
11	BY	9	313835	393935	1.00
11	3S	9	394410	432610	1.00
11	3S	13	492981	670681	0.93
11	3S	14	319549	425249	0.92
11	3S	15	496529	724429	0.85
12	3S	3	92477	207077	0.99
12	3S	5	6539	57439	0.99
12	BY	5	308100	465000	0.98
12	3S	7	932650	994350	0.89
12	BY	9	327892	393492	0.98
12	3S	9	395543	438943	0.99
12	BY	12	708936	741736	0.81
12	3S	13	531055	620455	0.92
12	3S	14	363239	471839	0.83
12	3S	15	317537	744837	0.89
13	3S	3	137844	219644	0.99
13	BY	4	1140684	1259184	0.95
13	3S	5	6535	76835	1.00
13	BY	5	326731	462131	0.99
13	3S	8	5198	123698	0.20
13	BY	9	344049	394149	0.99
13	3S	9	395108	432208	0.99
13	3S	13	518383	691383	0.94
13	3S	15	113329	195529	0.91
13	3S	15	465529	548929	0.84
14	3S	3	111173	217873	0.98
14	3S	5	1435	62835	1.00
14	BY	5	153019	226919	0.81
14	BY	5	193362	272662	0.83
14	BY	5	285062	467062	1.00
14	BY	5	337672	413672	0.98
14	BY	7	87383	142783	0.78
14	BY	7	100573	195573	0.85
14	3S	7	961406	1001106	0.80
14	3S	9	361620	432620	0.99
14	3S	13	514075	735675	0.99
14	3S	14	415078	483578	0.85

14	3S	15	49129	227029	0.93
14	BY	15	981009	1057509	0.99
15	3S	3	91977	211777	0.99
15	BY	4	962405	999505	0.80
15	3S	5	5335	72735	1.00
15	BY	5	139920	410720	0.99
15	BY	7	68009	257109	0.87
15	BY	10	394723	462323	0.81
15	3S	13	544486	684186	0.98
15	3S	14	374874	497074	0.90
15	3S	15	451405	713805	1.00
16	3S	3	100677	219177	0.99
16	3S	5	6639	43939	1.00
16	BY	5	143401	301901	0.95
16	BY	5	363002	457002	0.99
16	BY	7	99973	191773	0.88
16	3S	11	79889	366889	0.86
16	3S	13	567537	688837	1.00
16	3S	14	344174	524474	0.96
16	3S	15	126	221726	1.00
16	3S	15	449982	664382	1.00
17	3S	3	96650	211250	1.00
17	BY	4	1055912	1150512	0.93
17	BY	4	1169012	1240512	1.00
17	3S	5	6535	68135	1.00
17	BY	5	197225	297825	0.89
17	BY	5	323125	420925	1.00
17	BY	7	81340	142540	0.93
17	3S	8	59041	154741	0.07
17	3S	11	122072	372272	0.95
17	BY	12	643938	729338	0.93
17	3S	13	538573	681373	0.99
17	3S	14	374177	482077	0.94
17	3S	15	141726	218926	1.00
17	3S	15	491269	659569	0.96

Supplementary Table 3. Loci detected in control backcross pools. Start and stop positions were called as the 90% confidence interval around the point of maximum significance. Allele frequencies were calculated as the maximum within a 25-SNP window within a locus.

Segregant	Cross	Chromosome	Start	Stop	Frequency
2	3S	3	175351	222251	0.99
2	3S	5	6539	99439	1.00
2	BY	13	25470	83870	0.80
3	3S	3	95839	221139	0.99
3	3S	5	4935	352535	1.00
3	3S	16	451372	522072	0.85
4	3S	3	91477	228777	0.99
4	BY	4	1172722	1238422	0.82
4	3S	5	1335	79335	0.99
4	3S	14	319549	465949	0.97
5	3S	3	90277	226577	0.99
5	3S	3	157990	222790	0.99
5	BY	4	1095315	1264915	0.88
5	3S	5	6539	64439	0.99
5	3S	5	6539	64439	0.99
5	3S	14	312304	464204	0.98
5	3S	14	343473	427173	0.93
6	3S	3	105147	204847	0.97
6	3S	5	6539	89039	0.94
6	3S	14	324739	430039	0.91
7	3S	3	92213	221213	0.99
7	3S	5	1335	83635	0.99
7	3S	14	336535	428935	0.93
8	3S	3	70256	214056	1.00
8	3S	5	26335	97935	1.00
8	3S	14	354444	427244	0.95
9	3S	3	91468	220668	0.99
9	3S	4	127296	538696	0.83
9	3S	5	25139	78039	0.98
9	3S	14	341751	430651	0.93
10	3S	3	74057	218957	0.96
10	BY	4	1097984	1254184	0.86
10	3S	5	1335	117535	0.97
10	3S	14	312239	445239	0.93
11	3S	3	93677	219177	0.99

11	3S	5	1435	90435	1.00
11	3S	14	319649	441049	0.93
12	3S	3	91077	207077	0.99
12	BY	4	1091855	1221755	0.81
12	3S	5	6539	57439	1.00
12	3S	14	337139	441539	0.92
13	3S	3	92244	222244	0.99
13	BY	4	1171184	1255684	0.84
13	3S	5	1435	80735	1.00
14	3S	3	92073	222673	1.00
14	BY	4	1084535	1246235	0.82
14	3S	5	1435	59235	1.00
15	3S	3	91157	211857	0.99
15	3S	5	2835	78635	0.99
15	3S	14	313274	421374	0.95
15	BY	15	12077	98977	0.96
16	3S	3	100577	215577	0.99
16	BY	4	1095215	1220415	0.85
16	3S	5	6539	43939	1.00
16	3S	14	313274	424574	0.95
17	3S	3	104933	212933	0.99
17	BY	4	1172876	1214876	0.90
17	3S	5	6635	73435	1.00
17	3S	14	355180	412980	0.97

Supplementary Table 4. Predominant genotypes in mapping populations derived from each rough segregant.

Segregant	# loci detected	Predominant genotype
1	2	*FLO8*^{3S} *SSN8*^{mut}
2	3	*FLO8*^{3S} *MSS11*^{BY} *SSN8*^{mut}
3	4	*TRR1*^{3S} *FLO8*^{3S} *END3*^{3S} *SSN3*^{mut}
4	5	*FLO8*^{3S} *MSS11*^{BY} *END3*^{BY} *IRA2*^{mut} *SFL1*^{BY}
5	5	*IRA1*^{mut} *FLO8*^{3S} *XII-1*^{BY} *MSS11*^{BY} *END3*^{BY}
6	6	*IRA1*^{mut} *FLO8*^{3S} *XII-2*^{3S} *MSS11*^{BY} *END3*^{BY} *IRA2*^{3S}
7	10	*GPA2*^{3S} *FLO8*^{3S} *MDS3*^{3S} *FLO11*^{BY} *TPK3*^{BY} *MSS11*^{BY} *END3*^{BY} *IRA2*^{BY} *SFL1*^{BY} *GPB1*^{mut}
8	10	*GPA2*^{3S} *FLO8*^{3S} *MDS3*^{3S} *VIII*^{3S} *FLO11*^{BY} *IRC8*^{mut} *XIII*^{BY} *MSS11*^{BY} *IRA2*^{BY} *SFL1*^{BY}
9	5	*FLO8*^{3S} *FLO11*^{3S} *FLO11*^{BY} *MSS11*^{BY} *IRA2*^{BY}
10	6	*FLO8*^{3S} *FLO11*^{3S} *FLO11*^{BY} *TPK3*^{BY} *MSS11*^{BY} *IRA2*^{BY}
11	7	*GPA2*^{3S} *FLO8*^{3S} *MDS3*^{3S} *FLO11*^{3S} *FLO11*^{BY} *MSS11*^{BY} *SFL1*^{BY}
12	8	*FLO8*^{3S} *MGA1*^{BY} *FLO11*^{3S} *FLO11*^{BY} *XII-2*^{3S} *MSS11*^{BY} *END3*^{BY} *SFL1*^{BY}
13	7	*FLO8*^{3S} *VIII*^{3S} *FLO11*^{3S} *FLO11*^{BY} *MSS11*^{BY} *IRA2*^{BY} *SFL1*^{BY}
14	9	*GPA2*^{3S} *FLO8*^{3S} *MDS3*^{3S} *MGA1*^{BY} *FLO11*^{3S} *FLO11*^{BY} *MSS11*^{BY} *END3*^{BY} *IRA2*^{BY}
15	8	*IV*^{3S} *GPA2*^{3S} *FLO8*^{3S} *MDS3*^{3S} *X*^{3S} *MSS11*^{BY} *END3*^{BY} *SFL1*^{BY}
16	8	*GPA2*^{3S} *FLO8*^{3S} *MDS3*^{3S} *TPK3*^{BY} *MSS11*^{BY} *END3*^{BY} *IRA2*^{BY} *SFL1*^{BY}
17	10	*GPA2*^{3S} *FLO8*^{3S} *MDS3*^{3S} *VIII*^{3S} *TPK3*^{BY} *XII-2*^{3S} *MSS11*^{BY} *END3*^{BY} *IRA2*^{BY} *SFL1*^{BY}

Supplementary Table 5. Information regarding causal *de novo* mutations. Functional consequences were assessed by deleting the mutant allele from the rough segregant carrying a given lesion. The *IRA2* mutation with an asterisk refers to the one identified in Taylor et al. 2014. *PLOS Genetics*.

Mutant gene	Function of gene	Lesion	Functional consequence	Impact on gene sequence
GPB1	Multistep regulator of cAMP-PKA signaling	Single nucleotide mutation 2109G->T	Partial loss-of-function	Changes Leucine to Phenylalanine at codon 703. May disrupt Kelch domain[1]
IRA1	GTPase-activating protein; negatively regulates Ras	Single base deletion 1160ΔG	Null	Nonsense; Loss of Gpb2 binding site and GAP-related domain; Truncates 2694 amino acids, 87% of protein
IRA1	GTPase-activating protein; negatively regulates Ras	Single base deletion 7719ΔG	Null	Nonsense; Loss of Gpb2 binding site and disruption of GAP-related domain[2]; Truncates 503 amino acids, 16% of protein
IRA2	GTPase-activating protein; negatively regulates Ras	Single nucleotide mutation -60C->T	Null	Likely disrupts Tbp binding site[3]
*IRA2**	GTPase-activating protein; negatively regulates Ras	Single base deletion 8801ΔA	Partial loss-of-function	Nonsense; Loss of Gpb1 binding site[2]; Truncates 117 amino acids, 4% of protein
IRC8	Bud tip localized protein	Single base deletion 938ΔA	Null	Nonsense; Truncates 487 amino acids, 59% of protein
SSN3	Cyclin-dependent protein kinase; component of RNA pol II holoenzyme	Single base deletion 111ΔT	Null	Nonsense; Truncates 508 amino acids, 92% of protein
SSN8	Cyclin-like component of RNA polymerase II holoenzyme	22 base deletion 40Δ22	Null	Nonsense; Loss of cyclin box domain[4]; Truncates 291 amino acids, 90% of protein

Supplementary Table 6. SRA accession numbers for each pooled sequencing experiment.

RS1xBY4716	SAMN04126845
RS1x322134S	SAMN04126846
RS2xBY4716	SAMN04126847
RS2x322134S	SAMN04126848
RS3xBY4716	SAMN04126849
RS3x322134S	SAMN04126850
RS4xBY4716	SAMN04126851
RS4x322134S	SAMN04126852
RS5xBY4716	SAMN04126853
RS5x322134S	SAMN04126854
RS6xBY4716	SAMN04126855
RS6x322134S	SAMN04126856
RS7xBY4716	SAMN04126857
RS7x322134S	SAMN04126858
RS8xBY4716	SAMN04126859
RS8x322134S	SAMN04126860
RS9xBY4716	SAMN04126861
RS9x322134S	SAMN04126862
RS10xBY4716	SAMN04126863
RS10x322134S	SAMN04126864
RS11xBY4716	SAMN04126865
RS11x322134S	SAMN04126866
RS12xBY4716	SAMN04126867
RS12x322134S	SAMN04126868
RS13xBY4716	SAMN04126869
RS13x322134S	SAMN04126870
RS14xBY4716	SAMN04126871
RS14x322134S	SAMN04126872
RS15xBY4716	SAMN04126873
RS15x322134S	SAMN04126874
RS16xBY4716	SAMN04126875
RS16x322134S	SAMN04126876
RS17xBY4716	SAMN04126877
RS17x322134S	SAMN04126878
RS1xBY4716_control	SAMN04126879
RS1x322134S_control	SAMN04126880
RS2xBY4716_control	SAMN04126881
RS2x322134S_control	SAMN04126882
RS3xBY4716_control	SAMN04126883

RS3x322134S_control	SAMN04126884
RS4xBY4716_control	SAMN04126885
RS4x322134S_control	SAMN04126886
RS5xBY4716_control	SAMN04126887
RS5x322134S_control	SAMN04126888
RS6xBY4716_control	SAMN04126889
RS6x322134S_control	SAMN04126890
RS7xBY4716_control	SAMN04126891
RS7x322134S_control	SAMN04126892
RS8xBY4716_control	SAMN04126893
RS8x322134S_control	SAMN04126894
RS9xBY4716_control	SAMN04126895
RS9x322134S_control	SAMN04126896
RS10xBY4716_control	SAMN04126897
RS10x322134S_control	SAMN04126898
RS11xBY4716_control	SAMN04126899
RS11x322134S_control	SAMN04126900
RS12xBY4716_control	SAMN04126901
RS12x322134S_control	SAMN04126902
RS13xBY4716_control	SAMN04126903
RS13x322134S_control	SAMN04126904
RS14xBY4716_control	SAMN04126905
RS14x322134S_control	SAMN04126906
RS15xBY4716_control	SAMN04126907
RS15x322134S_control	SAMN04126908
RS16xBY4716_control	SAMN04126909
RS16x322134S_control	SAMN04126910
RS17xBY4716_control	SAMN04126911
RS17x322134S_control	SAMN04126912

Supplementary Table 7. Primers used in this study. For marked replacements, a gene of interest is amplified using primers GENE_mark1 and GENE_mark2. An MX cassette is amplified using primers universal3 and GENE_mark4. These amplicons are mixed together and transformed into a strain of interest. The two cassettes are joined by homologous recombination and replace the gene of interest, leaving behind a selectable MX cassette marker. Every mark2 primer begins with the sequence TAAATGTACGGGCGACAGTCACATCATGCCCCTGAGCTGCGCACGTCAAGACTGTCAAGG, which is homologous to all MX cassettes and allows for a recombination between the gene cassette and the MX cassette. Every mark4 primer ends with the sequence CGCACTTAACTTCGCATCTG, which allows for the amplification of an MX cassette.

The following primers can be used to generate allele swap strain using 'marked allele replacement', as in Matsui et al. 2015. *Genetics*. doi: 10.1534/genetics.115.180661.	
universal 3	CCTTGACAGTCTTGACGTGC
FLO11_coding_mark1	TCGCTTATTTGGTCCTTTCG
FLO11_coding_mark2	TAAATGTACGGGCGACAGTCACATCATGCCCCTGAGCTGCGCACGTCAAGACTGTCAAGGTTGCCAATGTATGAGAGTGG
FLO11_coding_mark4	CGTGTAAATCAAGTATGTCATTTCAGGAAACTTCCTCAATTCTTGGCGTACTATATTGAGCGCACTTAACTTCGCATCTG
FLO11_promoter_mark1	TGCGTATATGGATTTTTGAGG
FLO11_promoter_mark2	TAAATGTACGGGCGACAGTCACATCATGCCCCTGAGCTGCGCACGTCAAGACTGTCAAGG GCAGATGCAAACAAAAAGCA
FLO11_promoter_mark4	CACCACCACGATCGGAGAAGCGCTATTAGTAGCAATGGCTAAGCCTTGCCAGAACATGTAA CGCACTTAACTTCGCATCTG
GBP1_mark1	CCGTACCAATTCTTCTACAT
GBP1_mark2	TAAATGTACGGGCGACAGTCACATCATGCCCCTGAGCTGCGCACGTCAAGACTGTCAAGGCCCCGCGGAATTAATTAGTT
GBP1_mark4	GAAAAAATTTTCTCGTTTTCCTTTAGTCACTCTTGTCACATAAGGATTATCCGAACCCCGCGCACTTAACTTCGCATCTG
GPA2_mark1	AAGATATACCATATATTACG
GPA2_mark2	TAAATGTACGGGCGACAGTCACATCATGCCCCTGAGCTGCGCACGTCAAGACTGTCAAGGAACGGTTGTGCTTAATACAG
GPA2_mark4	CGGGATAATAACTATAATGACTACAATAATATAGTGGTATAACGCTATAAATTAAAAAATCGCACTTAACTTCGCATCTG

IRA1_mark1	GCCAATAAAATGATCAAAGG
IRA1_mark2	TAAATGTACGGGCGACAGTCACATCATGCCCCTGAGCTGCGCACGTCAAGACTGTCAAGGAAAACGTATATAATCACTGC
IRA1_mark4	AAAACAAAATATAATTATAAGGAAAAACGTATATAATCACTGCAATACTCTAATTTAAAACGCACTTAACTTCGCATCTG
IRA2_mark1	GGACATGCTTCTCCCTGAAG
IRA2_mark2	TAAATGTACGGGCGACAGTCACATCATGCCCCTGAGCTGCGCACGTCAAGACTGTCAAGGTACCCTTGTAAGTGTCATCC
IRA2_mark4	GCACAGATCCCAGAGAAAAGCAGGGAAACAAGAAAATAAGAAAACAAGAAAAACAGTAGTCGCACTTAACTTCGCATCTG
IRC8_mark1	GCAAAACGCACATACCCACA
IRC8_mark2	TAAATGTACGGGCGACAGTCACATCATGCCCCTGAGCTGCGCACGTCAAGACTGTCAAGGATATGAATAAGTCGGTTGGT
IRC8_mark4	ATCGTGTTCTACGGGATCAAAATAGTTGCTTTTAGCAGTTCCCATAGGTATCTTTGATACCGCACTTAACTTCGCATCTG
MDS3_mark1	GTGGCTAAGGCAGACTCCGT
MDS3_mark2	TAAATGTACGGGCGACAGTCACATCATGCCCCTGAGCTGCGCACGTCAAGACTGTCAAGGGTGCGAGTAACTATCCTGGG
MDS3_mark4	GGAAGCAATCCGTTTTGAGATGTATAGCAGCATATTCTTGGATTATTAAGAACTTTATATCGCACTTAACTTCGCATCTG
SSN3_mark1	TGTGGCTTAAGTTGCGTTTC
SSN3_mark2	TAAATGTACGGGCGACAGTCACATCATGCCCCTGAGCTGCGCACGTCAAGACTGTCAAGGCTATCTTCTGTTTTTCTTTC

SSN3_mark4	GAATATAATAGTGACAGTGCTGTGGAATGAAAAATTCCAAATATATATAAAAATAGAAGCCGCACTTAACTTCGC ATCTG
SSN8_mark1	CTGAATCTCAAAAGTTAGAC
SSN8_mark2	TAAATGTACGGGCGACAGTCACATCATGCCCCTGAGCTGCGCACGTCAAGACTGTCAAGGGTTTTTAAATTTAT TCTTCG
SSN8_mark4	GACGAAACATTTCCAAAACGGATCATCACCACCATAATGATTGAATTTACAGGCTTAACGCGCACTTAACTTCGC ATCTG
TPK3_mark1	AGACACTTTGTGCAGTCGTC
TPK3_mark2	TAAATGTACGGGCGACAGTCACATCATGCCCCTGAGCTGCGCACGTCAAGACTGTCAAGGATGGCGTATATGA ATGCTCC
TPK3_mark4	GAAAAGTCCTAGATCACTTTGAACGTCCCAGTCTTCTGAGGACGCAAATGTAGTCACAATCGCACTTAACTTCGC ATCTG
The following primers can be used to amplify the CORE cassette (Storici et al. 2001. Nature Biotechnology. 19: 773-776) for targeted gene deletion.	

gpb1Δ_F	TTTCTCGTTTTCCTTTAGTCACTCTTGTCACATAAGGATTATCCGAACCCCGCCCCGCGGGAGCTCGTTTTCGACACTGG
gpb1Δ_R	TGAGCCGACCTCCCTATATCGGCTACTTTAAGGCTTTCCGTACCAATTCTTCTACATAAGTCCTTACCATTAAGTTGATC
ira1Δ_F	TTAGGAGCACGACATTCTTGCCAGTATCATTGTTGCTAATCTTTTTCTCTCATAAATTGCTCCTTACCATTAAGTTGATC
ira1Δ_R	GTTAAGCTATTTAACGAAAGCGTATAAAGTCAAGTGATCATCTTTTGCCCTGCAAATAGAGAGCTCGTTTTCGACACTGG
ira2Δ_F	GCATATAGCATTGTCCTCTGTTATTCGTTTTGCTTTTCTCCTTTAGTGTTACTTTTCCCCCAACGGAGCTCGTTTTCGACACTGG
ira2Δ_R	ATGTACATTCATGCTTACAGATAGATATTGATATTTCTTTCATTAGTTTATGTAACACCTTCCTTACCATTAAGTTGATC
irc8Δ_F	CTTCTTACGTATCAGAACAAGAAAGCATTTCCAAAGTAATTGCATTTGCCCTTGAGCAGTGAGCTCGTTTTCGACACTGG
irc8Δ_R	TTAATGAAATAAATCGTGTTCTACGGGATCAAAATAGTTGCTTTTAGCAGTTCCCATTCCTTACCATTAAGTTGATC
ssn3Δ_F	GAATATAATAGTGACAGTGCTGTGGAATGAAAAATTCCAAATATATATAAAAATAGAAGCTCCTTACCATTAAGTTGATC
ssn3Δ_R	AAAGGTTTATAGGAAAGAAAAAAGGCGGAAGGGTATACTGAAGTTAGTAATTTTGCTTCCGAGCTCGTTTTCGACACTGG
ssn8Δ_F	ATCATCACCACCATAATGATTGAATTTACAGGCTTAACGGTTTTTAAATTTATTCTTCGCGAGCTCGTTTTCGACACTGG
ssn8Δ_R	AAATGCCCTCTCAAACTTTAGTTGAAGAGCGATAAGGCATCTGAATCTCAAAAGTTAGACTCCTTACCATTAAGTTGATC

Supplementary Note 1. Aneuploidies and chromosomal rearrangements do not contribute to our current results. To determine if aneuploidies caused rough morphology in any individuals, the average coverage of each chromosome was examined in the rough and control backcross pools. Only mapping populations descended from rough segregant 12 possessed an aneuploidy, which occurred on Chromosome I. 38 and 34% enrichment of this chromosome was observed relative to average genome-wide coverage in the BY and 3S backcrosses, respectively. However, similar enrichment in coverage for Chromosome I was seen in control pools descended from rough segregant 12, suggesting that the aneuploidy does not contribute to the rough phenotype. Furthermore, analyses aimed at identifying chromosomal rearrangements failed to identify any such events.

Supplementary References

1 Phan, V. T. *et al.* The RasGAP proteins Ira2 and neurofibromin are negatively regulated by Gpb1 in yeast and ETEA in humans. *Mol Cell Biol* **30**, 2264-2279 (2010).

2 Harashima, T., Anderson, S., Yates, J. R., 3rd & Heitman, J. The kelch proteins Gpb1 and Gpb2 inhibit Ras activity via association with the yeast RasGAP neurofibromin homologs Ira1 and Ira2. *Mol Cell* **22**, 819-830 (2006).

3 Venters, B. J. *et al.* A comprehensive genomic binding map of gene and chromatin regulatory proteins in Saccharomyces. *Mol Cell* **41**, 480-492 (2011).

4 Kuchin, S., Yeghiayan, P. & Carlson, M. Cyclin-dependent protein kinase and cyclin homologs SSN3 and SSN8 contribute to transcriptional control in yeast. *Proc Natl Acad Sci U S A* **92**, 4006-4010 (1995).
